# Gonadotropin Releasing Hormone agonist (GnRHa) during chemotherapy and post-cancer childbirths – a Nationwide population-based cohort study of 24,922 women diagnosed with cancer in Sweden

**DOI:** 10.1016/j.eclinm.2023.102335

**Published:** 2023-12-07

**Authors:** Kenny A. Rodriguez-Wallberg, Helle Kieler, Theodoros Foukakis, Jiong Li, Mika Gissler, Anna Sara Oberg, Jonas Bergh, Frida E. Lundberg

**Affiliations:** a Department of Oncology-Pathology, Karolinska Institutet, Stockholm, Sweden; b Division of Gynecology and Reproduction, Department of Reproductive Medicine, Karolinska University Hospital, Stockholm, Sweden; c Laboratory of Translational Fertility Preservation, BioClinicum, New Karolinska University Hospital, Stockholm, Sweden; d Department of Medicine, Centre for Pharmacoepidemiology (CPE), Karolinska Institutet, Stockholm, Sweden; e Breast Cancer Center, Theme Cancer, Karolinska Comprehensive Cancer Center, Karolinska University Hospital, Stockholm, Sweden; f Department of Clinical Medicine-Department of Clinical Epidemiology, Aarhus University Hospital, Aarhus, Denmark; g Department of Knowledge Brokers, THL Finnish Institute for Health and Welfare, Helsinki, Finland; h Department of Medical Epidemiology and Biostatistics, Karolinska Institutet, Stockholm, Sweden; i Region Stockholm, Academic Primary Health Care Centre, Stockholm, Sweden; j Department of Molecular Medicine and Surgery, Karolinska Institutet, Stockholm, Sweden

**Keywords:** Cancer treatment, Gonadal toxicity, Iatrogenic infertility, Fertility preservation, Ovarian suppression, GnRHa, Female, Breast cancer, Lymphoma, Live birth

## Abstract

**Background:**

Worldwide, an increasing number of women with cancer are receiving Gonadotropin Releasing Hormone agonist (GnRHa) co-treatment during chemotherapy aiming at ovarian protection. There is divergence among guidelines, and some have recommended GnRHa co-treatment for women with breast cancer, however, the effect of GnRHa on future fertility is uncertain.

**Methods:**

In this population-based cohort study we included all women diagnosed with cancer at ages 15–45 between July 2005 and March 2017 in Sweden, identified in the Swedish Cancer Register. Exposure to GnRHa co-treatment was captured using the Prescribed Drug Register. Post-cancer childbirth, extracted from the Medical Birth Register, was the main outcome. Secondary outcomes included childbirths achieved through natural conception (NC), infertility diagnosis and cancer mortality. For each outcome, adjusted hazard ratios (aHR) and 95% confidence intervals (CI) were estimated using delayed-entry Cox models, stratified by age and cancer site.

**Findings:**

Among 24,922 women diagnosed with cancer, 1.5% had GnRHa co-treatment. Breast cancer diagnoses were found in 80.2% of GnRHa exposed women and the GnRHa exposure was not associated with higher rates of childbirth (aHR 1.23, 95% CI 0.80–1.89), or NC childbirth (aHR 1.02, 95% CI 0.62–1.67), whereas the rate of infertility was significantly higher (aHR 2.42, 95% CI 1.44–4.08). In women with lymphoma and other cancers, GnRHa exposure was not associated with higher rates of childbirth, NC childbirth or infertility. GnRHa exposure was not associated with higher cancer mortality for any cancer type.

**Interpretation:**

We did not find evidence of improved or maintained fertility, estimated as childbirth rates post-cancer, in women who received GnRHa during cancer treatment.

**Funding:**

This study was financed by research grants from The 10.13039/501100002794Swedish Cancer Society (CAN 2017/704; 190249Pj, 200170F), The 10.13039/501100004359Swedish Research Council (Dnr 2019-00446), the Nordic Cancer Union NCU (Grant 2017), The Swedish Childhood Cancer Fund (KP2016-0031), 10.13039/501100007232Radiumhemmets Forskningsfonder (Dnr: 201313), 10.13039/501100004348Stockholm County Council (FoUI-953912) and 10.13039/501100004047Karolinska Institutet (Dnr 2020-01963).

**Research in context**

**Evidence before this study**

The use of GnRH agonists for ovarian protection during chemotherapy, particularly in women with breast cancer, is widely spread and increasing. This is supported by small randomized clinical trials of women with breast cancer showing a reduced prevalence of amenorrhea, with a plausible effect on fertility after a short follow-up. Although randomized, all available studies have been unblinded, and there is evidence that the women who received the GnRHa co-treatment in the studies have attempted pregnancy more frequently than the women that did not receive the GnRHa co-treatment. On the other hand, randomized studies that have measured biochemical markers of ovarian reserve after breast cancer treatment have not found any protection in the groups receiving GnRHa co-treatment. Additionally, a randomized study of women with lymphoma with 7-years follow-up did not find any protection in ovarian markers or in pregnancies achieved post-treatment.

**Added value of this study**

Using Swedish population-based registers, we present real world evidence of the probability of post-cancer live birth with or without GnRHa co-treatment in a large Nationwide cohort of women 15–45 years of age at time of cancer treatment.

**Implications of all the available evidence**

Our findings do not support an association of receiving GnRHa at time of cancer treatment with an increased likelihood of achieving post-cancer births. There is an urgent need of well-designed and well-powered randomized controlled trials on this research question, and these studies should ideally be placebo-controlled and double blinded, to finally evaluate the efficacy of GnRHa co-treatment during chemotherapy as fertility protective in women treated for cancer.

## Introduction

Since temporary ovarian suppression during chemotherapy was hypothesized to protect fertility in the 1980s,^1^ Gonadotropin Releasing Hormone agonists (GnRHa) have become commonly prescribed for fertility protection and this treatment has been recently recommended by the European St. Gallen International Consensus guidelines for women with breast cancer^2^ and the ESMO BCY5 Consensus guideline for breast cancer in young women.^3^ However, results from randomized clinical trials (RCTs) have so far been inconclusive.^4^^,^^5^ While studies in women with breast cancer seem to indicate a beneficial effect of GnRHa in resumption of ovarian function post-chemotherapy^6^ and a plausible effect on fertility,^7^ no significant effects have been evidenced on biochemical markers of ovarian reserve.^8^ In women with lymphoma no benefit could be found regarding either ovarian reserve markers or reproductive outcomes.^5^ The data combined in a meta-analysis of women with breast cancer support a reduction of premature ovarian insufficiency rate and a higher frequency of pregnancies.^6^ However, findings in a meta-analysis including data on biochemical markers of ovarian reserve during and after chemotherapy do not support a protective effect.^9^ Although current guidelines provided by the American Society of Clinical Oncology, ASCO, and the European Society of Human Reproduction and Endocrinology (ESHRE) state that GnRHa should not substitute the use of recognized effective methods for fertility preservation for female cancer patients,^10^^,^^11^ GnRHa is being widely prescribed to adult and adolescent women in various clinical situations, with the aim to preserve their future fertility.^12^^,^^13^

In the current study, we used the Swedish population-based registers to investigate the association between GnRHa dispensation at time of cancer treatment and the childbirth rate post cancer diagnosis among women with breast cancer, lymphoma, and other cancers.

## Methods

### Study population

The Swedish Cancer Register was used to identify all women aged 15–45 years diagnosed with any cancer between July 1st 2005 and March 31st 2017 (n = 26,938), with a potential follow-up of at least 9 months at the time of data extraction. Using the unique personal identity number assigned to all Swedish citizens and residents, individuals were linked to several other population-based registers. Exclusions were done due to previous cancers before start of follow-up (n = 627), women with breast cancer who received tamoxifen within 120 days of diagnosis (n = 1366) indicating absence of chemotherapy treatment and reused personal identity numbers (n = 23). The final study population included 24,922 women. First incident primary malignant tumors were grouped by organ system using the 10th version of *International Statistical Classification of Diseases and Related Health Problems* (ICD-10) codes C00–C99 (Appendix p 2). Information on tumor size, lymph node involvement and distant metastases was used to define stage at diagnosis for breast cancer patients. This study followed the Strengthening the Reporting of Observational Studies in Epidemiology (STROBE) guidelines.

### Exposure

The Prescribed Drug Register was used to determine exposure to GnRHa, which was defined as at least one dispensation of GnRHa (*Anatomical Therapeutic Chemical Classification System* [ATC] codes starting with L02AE) between 30 days before and 180 days after date of cancer diagnosis. Women with a cancer diagnosis who were not dispensed GnRHa during this period were considered unexposed. Tamoxifen treatment (ATC L02BA) between 4 months and 5 years of breast cancer diagnosis was used to define use of adjuvant endocrine therapy. Breast cancer patients who only received GnRHa concomitantly with tamoxifen as adjuvant endocrine therapy were considered unexposed (n = 44).

### Outcomes

Information on childbirth was retrieved from the Medical Birth Register.^14^ The first live birth occurring more than 9 months after cancer diagnosis was used as the outcome in models of childbirth. For these births, the mothers’ previous parity and obstetric and perinatal data were also extracted. Any diagnosis of infertility (ICD-10 N97) registered in the National Patient Register at least 9 months after cancer diagnosis was used as the outcome in models of infertility. Causes and date of death were identified using the Cause of Death Register, and death due to any cancer (ICD-10 C00–C99) was used as the outcome in models of cancer mortality.

Information on use of assisted reproductive technology (ART) treatments resulting in a live birth was obtained from the National Board of Health and Welfare up to 2006 and from the National Quality Registry of Assisted Reproduction for 2007–2017. Childbirths occurring without the use of ART were defined as occurring through natural conception (NC childbirth).

The highest attained education level in the year of cancer diagnosis was retrieved from the Swedish Education Register, and country of birth from the Register of the Total Population. The Prescribed Drug Register was also used to identify the women who received gonadotropin stimulation treatments (ATC G03GA02-06 or 09) in combination with GnRH antagonists (ATC H01CC) for fertility preservation between 30 days before and 180 days after date of cancer diagnosis, which is the standard treatment protocol used for fertility preservation aimed at cryopreservation of embryos or oocytes before initiation of cancer treatment.^15^

### Statistical analysis

The distribution of baseline characteristics of all women, and obstetric and perinatal outcomes for the first post-cancer birth, were compared between women exposed and unexposed to GnRHa using Pearson's chi-square test.

Hazard ratios (HR) and 95% confidence intervals (CI) for any childbirth, ART and NC childbirth, diagnosis of post-cancer infertility and cancer mortality were estimated using delayed-entry stratified Cox proportional hazard models, with time since diagnosis as the underlying time scale. Since the main outcome was childbirth, the date of entry was 9 months after cancer diagnosis, aiming to capture only pregnancies conceived after the cancer was diagnosed. Separate models were fitted for breast cancer, lymphoma, and other cancer diagnoses. All models were stratified by age at diagnosis (1-year categories) and cancer site (according to Appendix p 2). Person-years at risk were accrued from date of entry until the date of the event of interest or censored at death (other cause of death for models of cancer mortality), emigration, new cancer diagnosis or end of follow-up (December 31st, 2017), whichever occurred first. Time at-risk of post-cancer infertility was additionally censored at date of live birth. Adjusted models included relevant confounders for the investigation of cancer and of fertility outcomes, categorized according to Table 1; country of birth, calendar period based on year of cancer diagnosis, parity at diagnosis and education level achieved before diagnosis. For breast cancer, stage at diagnosis was included in additional adjusted models, categorized according to Table 2. Multiple imputation using chained equations was used for missing values of education and breast cancer stage. Both variables were assumed to be missing at random, given the other predictors in the imputation models: GnRHa exposure, age at diagnosis (linear), calendar period, parity, country of birth, adjuvant endocrine therapy, and the cumulative hazards of childbirth and cancer death (from Nelson-Aalen), as well as education and stage respectively. Ten cycles of chained equations were used, and results from 50 imputation cycles were combined by Rubin's rules. The same approach was used to impute education level for the analysis of other cancers (not considering stage or adjuvant therapy). As sensitivity analyses, we repeated the analyses among women who were nulliparous at diagnosis, among women ages 15–39 years at diagnosis, and in breast cancer patients stratified by adjuvant endocrine therapy. The proportional hazards assumption was evaluated using the Schoenfeld residuals from the adjusted models. Data management and analyses were performed using Stata 17/BE (Stata Corp LLC.). All statistical tests were two-sided with a significance level of 5%. No adjustments were made for multiple comparisons, hence the 95% confidence intervals around the HRs may not be reproducible.

**Table 1:** Characteristics of women diagnosed with cancer at ages 15–45 years in Sweden 2005–2017.

	Women without GnRHa (unexposed)		Women with GnRHa (exposed)		Total women in the cohort		p-value
	No.	%	No.	%	No.	%	
**Number of patients**	24,539	100.0	383	100.0	24,922	100.0	
**Cancer site**							<0.001
Breast	6639	27.1	307	80.2	6946	27.9	
Skin	3969	16.2	1	0.3	3970	15.9	
Endocrine glands	3352	13.7	8	2.1	3360	13.5	
Cervix	2562	10.4	7	1.8	2569	10.3	
Other gynecological	1254	5.1	2	0.5	1256	5.0	
Digestive organs	1724	7.0	6	1.6	1730	6.9	
Central nervous system	1609	6.6	2	0.5	1611	6.5	
Lymphoma	1112	4.5	32	8.4	1144	4.6	
*Hodgkin lymphoma*	*537*	*2.2*	*25*	*6.5*	*562*	*2.3*	
*Other lymphoma*	*575*	*2.3*	*7*	*1.8*	*582*	*2.3*	
Leukemia	582	2.4	8	2.1	590	2.4	
Respiratory and intrathoracic	406	1.7	4	1.0	410	1.6	
Urinary tract	335	1.4	2	0.5	337	1.4	
Mesothelial and soft tissue	274	1.1	1	0.3	275	1.1	
Bone and cartilage	109	0.4	3	0.8	112	0.4	
Other cancers	612	2.5	0	0.0	612	2.5	
**Age at cancer diagnosis**							<0.001
15–19	734	3.0	9	2.3	743	3.0	
20–24	1288	5.2	20	5.2	1308	5.2	
25–29	2288	9.3	81	21.1	2369	9.5	
30–34	3652	14.9	140	36.6	3792	15.2	
35–39	5553	22.6	71	18.5	5624	22.6	
40–45	11,024	44.9	62	16.2	11,086	44.5	
**Country of birth**							0.056
Nordic	20,385	83.1	304	79.4	20,689	83.0	
Non-Nordic	4154	16.9	79	20.6	4233	17.0	
**Parity at cancer diagnosis**							<0.001
0 (nulliparous)	8095	33.0	222	58.0	8317	33.4	
1 child	4429	18.0	84	21.9	4513	18.1	
≥2 children	12,015	49.0	77	20.1	12,092	48.5	
**Year of cancer diagnosis**							<0.001
2005–2008	6562	26.7	81	21.1	6643	26.7	
2009–2011	6199	25.3	54	14.1	6253	25.1	
2012–2014	6655	27.1	102	26.6	6757	27.1	
2015–2017	5123	20.9	146	38.1	5269	21.1	
**Education before cancer diagnosis**							0.001
Compulsory school	2585	10.5	31	8.1	2616	10.5	
Secondary school	10,312	42.0	137	35.8	10,449	41.9	
Higher education <3 y	3581	14.6	52	13.6	3633	14.6	
Higher education ≥3 y	7065	28.8	149	38.9	7214	28.9	
Missing	996	4.1	14	3.7	1010	4.1	
**Fertility preservation aimed at oocyte or embryo cryopreservation**^a^	478	1.9	178	46.5	656	2.6	<0.001

Abbreviations: GnRHa = Gonadotropin-releasing hormone agonists.

a By use of gonadotropins and GnRH antagonists.

**Table 2:** Characteristics of women diagnosed with breast cancer at ages 15–45 years in Sweden 2005–2017.

	Women without GnRHa (unexposed)		Women with GnRHa (exposed)		Total women in the cohort		p-value
	No.	%	No.	%	No.	%	
**Number of patients**	6639	100.0	307	100.0	6946	100.0	
**Age at cancer diagnosis**							<0.001
15–19 years	4	0.1	0	0.0	4	0.1	
20–24 years	33	0.5	7	2.3	40	0.6	
25–29 years	200	3.0	60	19.5	260	3.7	
30–34 years	712	10.7	117	38.1	829	11.9	
35–39 years	1635	24.6	64	20.8	1699	24.5	
40–45 years	4055	61.1	59	19.2	4114	59.2	
**Country of birth**							0.35
Nordic	5355	80.7	241	78.5	5596	80.6	
Non-Nordic	1284	19.3	66	21.5	1350	19.4	
**Parity at cancer diagnosis**							<0.001
0 (nulliparous)	1384	20.8	164	53.4	1548	22.3	
1 child	1223	18.4	73	23.8	1296	18.7	
≥2 children	4032	60.7	70	22.8	4102	59.1	
**Year of cancer diagnosis**							<0.001
2005–2008	1894	28.5	66	21.5	1960	28.2	
2009–2011	1734	26.1	39	12.7	1773	25.5	
2012–2014	1706	25.7	81	26.4	1787	25.7	
2015–2017	1305	19.7	121	39.4	1426	20.5	
**Education before cancer diagnosis**							0.026
Compulsory school	505	7.6	22	7.2	527	7.6	
Secondary school	2768	41.7	107	34.9	2875	41.4	
Higher education <3 y	1050	15.8	43	14.0	1093	15.7	
Higher education ≥3 y	2160	32.5	127	41.4	2287	32.9	
Missing	156	2.3	8	2.6	164	2.4	
**Adjuvant endocrine therapy**							0.002
Yes	4140	62.4	164	53.4	4304	62.0	
No	2499	37.6	143	46.6	2642	38.0	
**Breast cancer stage at diagnosis**^b^							0.14
0	338	5.1	6	2.0	344	5.0	
I	2147	32.3	93	30.3	2240	32.2	
II	2208	33.3	109	35.5	2317	33.4	
III	1244	18.7	64	20.8	1308	18.8	
IV	119	1.8	4	1.3	123	1.8	
Missing	583	8.8	31	10.1	614	8.8	
**Fertility preservation aimed at oocyte or embryo cryopreservation**^a^	265	4.0	145	47.2	410	5.9	<0.001

Abbreviations: GnRHa = Gonadotropin-releasing hormone agonists.

a By use of gonadotropins and GnRH antagonists.

b Stage information mainly based on clinical examination causing a high proportion of stage 0.

### Ethics statement

Ethical approval for the study was granted by the Regional Ethics Committee of Stockholm (Dnr. 2013/1849-31/2, 2018/386-32 and 2019-01529). The requirement for informed consent was waived with the authorization of the ethical committee because of the observational nature of the study.

### Role of the funding source

The funding source had no role in design of the study, in the collection, analysis or interpretation of data, in the writing of the report or in the decision to submit the paper for publication. KARW and FEL have directly accessed and verified the underlying data reported, and had final responsibility to publish the study findings.

## Results

Among the 24,922 women included in the study, the prevalence of GnRHa use in connection with cancer treatment was low across all diagnoses (n = 383, 1.5%). The mean age at cancer diagnosis was 32.7 years in women who received GnRHa treatment and 36.6 years in unexposed women. The mean follow-up time was 4.7 and 5.8 years from diagnosis in women exposed and unexposed to GnRHa, respectively, and GnRHa co-treatments became more frequent over time. The proportion of patients receiving fertility preservation was 2.6% in the total cohort (n = 656), with significantly higher use in the group of women with GnRHa dispensations.

Table 1 summarizes demographic and clinical characteristics of the full study cohort. The most common cancers diagnosed in the cohort were breast (n = 6946, 27.9%), skin (n = 3970, 15.9%) and endocrine gland cancers (n = 3360, 13.5%). Breast cancer diagnosis accounted for 80.2% of all GnRHa dispensations (n = 307) and lymphomas for 8.4% (n = 32). Women who received GnHRa were younger and more often nulliparous at diagnosis and had attained a higher level of education. Fertility preservation treatments aimed at oocyte or embryo cryopreservation were identified in 46.5% of women who also received GnRHa treatment (n = 178) and 1.9% of women who did not (n = 478).

Characteristics of breast cancer patients are presented in Table 2. Breast cancers were diagnosed at a mean age of 33.9 years in women who received GnRHa and at 39.8 years in unexposed women. The mean follow-up time was 4.6 years from diagnosis of breast cancer in exposed and 6.0 years in unexposed women, respectively. In total, 4.4% of breast cancer patients had dispensations of GnRHa in connection with cancer treatment (n = 307). Women who received GnRHa were younger and more often nulliparous at time of breast cancer diagnosis, and more likely had attained a higher education level. Also, women exposed to GnRHa were less likely to receive adjuvant endocrine therapy, while the distribution of breast cancer stage at diagnosis was similar among exposed and unexposed. Nearly half (n = 145, 47.2%) of women with GnRHa dispensations also underwent fertility preservation treatments with gonadotropins and GnRH antagonists, compared to 4.0% of women without GnRHa (n = 265). The distribution of baseline characteristics for patients with lymphoma and other cancers are presented separately in Appendix p 3 and p 4, respectively.

### Rates of childbirth, infertility and mortality after cancer

Rates of any childbirth, NC childbirth, infertility and cancer mortality in the cohort are presented in Table 3. In the unadjusted models stratified by age, women with breast cancer and GnRHa co-treatment had a higher childbirth rate after cancer (HR 1.67, 95% CI 1.10–2.54). After adjusting for previous parity, country of birth, calendar period, education and cancer stage, the HR of childbirth was 1.23 (0.80–1.89). Parity at cancer diagnosis accounted for most of this attenuation, as nulliparous women tended to have more births during follow-up. The corresponding HRs of NC childbirth were 1.31 (0.81–2.12) when stratifying by age only and 1.02 (0.62–1.68) after adjustment, respectively. Women with breast cancer who received GnRHa had a significantly higher rate of receiving infertility diagnoses after cancer treatment (adjusted HR 2.42, 1.44–4.07), while cancer mortality rates did not differ from that of unexposed women (adjusted HR 1.08, 0.72–1.60).

**Table 3:** Rates of any childbirth, naturally conceived (NC childbirth), infertility and cancer mortality in women diagnosed with cancer at ages 15–45 years in Sweden 2005–2017.

	With GnRHa		No GnRHa (ref)		Model 1	Model 2	Model 3
	Events	PY	Events	PY	HR (95% CI)	HR (95% CI)	HR (95% CI)
**Breast cancer**							
Childbirth	28	1076	168	33,125	1.67 (1.10–2.54)	1.26 (0.82–1.93)	1.23 (0.80–1.89)
NC childbirth	20	1076	148	33,125	1.31 (0.81–2.12)	1.05 (0.64–1.71)	1.02 (0.62–1.68)
Infertility	24	1030	64	32,943	4.15 (2.51–6.87)	2.40 (1.43–4.00)	2.42 (1.44–4.07)
Cancer mortality	28	1158	634	33,792	1.09 (0.73–1.61)	1.07 (0.72–1.59)	1.08 (0.72–1.60)
**Lymphoma**							
Childbirth	8	99	208	5112	1.35 (0.64–2.86)	1.24 (0.58–2.66)	–
NC childbirth	8	99	194	5112	1.49 (0.70–3.18)	1.40 (0.65–3.00)	–
Infertility	2	94	59	4943	1.41 (0.31–6.35)	1.28 (0.26–6.29)	–
Cancer mortality	0	142	41	5943	NA	NA	–
**Other cancers than breast and lymphoma**							
Childbirth	7	135	1988	70,310	1.29 (0.59–2.82)	1.31 (0.60–2.87)	–
NC childbirth	3	135	1817	70,310	0.56 (0.17–1.78)	0.59 (0.18–1.89)	–
Infertility	5	118	492	69,060	2.55 (0.94–6.92)	2.42 (0.89–6.58)	–
Cancer mortality	5	176	1419	78,447	0.71 (0.27–1.84)	0.71 (0.27–1.85)	–

Model 1 stratified by age at diagnosis and cancer type, adjusted for time since diagnosis.

Model 2 further adjusted for country of birth, education level, calendar period, and parity before diagnosis.

Model 3 further adjusted for breast cancer stage at diagnosis.

Abbreviations: GnRHa = Gonadotropin-releasing hormone agonists, NC = natural conception.

In women with lymphoma, GnRHa treatment was not significantly associated with either childbirth rate (adjusted HR 1.24, 0.58–2.66) or infertility diagnosis (adjusted HR 1.28, 0.26–6.29), and there were no childbirths conceived using ART or cancer deaths among women with GnRHa (Table 3). In women with cancers other than breast or lymphoma, the adjusted HR for childbirth was 1.31 (0.60–2.87), for NC childbirth 0.59 (0.18–1.89), for post-cancer infertility diagnosis 2.42 (0.89–6.58) and for cancer mortality 0.71 (0.81, 0.27–1.85) when comparing women exposed and unexposed to GnRHa co-treatment.

In analyses including only women who were nulliparous at time of cancer diagnosis, the estimated HRs for childbirth were higher but still remained statistically non-significant (Appendix p 5). In nulliparous women diagnosed with breast cancer and treated with GnRHa, the adjusted HR for childbirth was 1.46 (0.86–2.47). In analyses including only women up to 39 years of age at cancer diagnosis (Appendix p 6), results were similar to those presented in Table 3. Among breast cancer patients who received adjuvant endocrine therapy, the estimated HRs for childbirth were higher but statistically non-significant (Appendix p 7). The adjusted HR for childbirth in GnRHa co-treated women was 1.56 (0.77–3.16) in the group with endocrine therapy, and 1.12 (0.55–2.26) in the group without endocrine therapy.

### Characteristics of first childbirth after cancer

Among the 2407 women who gave birth after cancer diagnosis, 43 (1.8%) had received GnRHa treatment (Table 4). There were no significant differences in age at delivery or time since cancer diagnosis between exposed and unexposed women. Around half (23/43, 53.5%) of women exposed to GnRHa had also received fertility preservation at the time of cancer diagnosis and 20 (46.5%) had been diagnosed with infertility before giving birth. Conception through ART treatment was more common among GnRHa exposed women (12/43, 27.9%) than among unexposed (94/2364, 8.7%). There were no significant differences in pregnancy complications, mode of delivery or adverse birth outcomes between the groups. In women who gave birth following a breast cancer diagnosis (Appendix p 8), preterm birth was slightly more common in women with breast cancer exposed to GnRHa (5/28, 17.9%) compared to unexposed women (10/168, 6.0%).

**Table 4:** Characteristics at first childbirth achieved after cancer diagnosis in women who received a diagnosis of cancer at ages 15–45 years in Sweden 2005–2017.

	Women without GnRHa (unexposed)		Women with GnRHa (exposed)		Total		p-value
	No.	%	No.	%	No.	%	
**Women with childbirth**	2364	100.0	43	100.0	2407	100.0	
**Previous cancer diagnosis**							<0.001
Breast	168	7.1	28	65.1	196	8.1	
Skin	576	24.4	1	2.3	577	24.0	
Endocrine glands	673	28.5	3	7.0	676	28.1	
Cervix	203	8.6	1	2.3	204	8.5	
Other gynecological	81	3.4	0	0.0	81	3.4	
Digestive organs	98	4.1	1	2.3	99	4.1	
Central nervous system	170	7.2	0	0.0	170	7.1	
Lymphoma	208	8.8	8	18.6	216	9.0	
*Hodgkin's lymphoma*	*133*	*5.6*	*6*	*14.0*	*139*	*5.8*	
*Other lymphoma*	*75*	*3.2*	*2*	*4.7*	*77*	*3.2*	
Leukemia	31	1.3	1	2.3	32	1.3	
Respiratory and intrathoracic	16	0.7	0	0.0	16	0.7	
Urinary tract	24	1.0	0	0.0	24	1.0	
Mesothelial and soft tissue	36	1.5	0	0.0	36	1.5	
Bone and cartilage	17	0.7	0	0.0	17	0.7	
Other cancers	63	2.7	0	0.0	63	2.6	
**Age at time of delivery**							0.12
<20 years	2	0.1	0	0.0	2	0.1	
20–29 years	664	28.1	6	14.0	670	27.8	
30–39 years	1523	64.4	31	72.1	1554	64.6	
≥40 years	175	7.4	6	14.0	181	7.5	
**Time between cancer diagnosis and birth**							0.56
<2 years	730	30.9	10	23.3	740	30.7	
2–4 years	1136	48.1	23	53.5	1159	48.2	
≥5 years	498	21.1	10	23.3	508	21.1	
**Previous parity before cancer diagnosis**							0.10
0 (nulliparous)	1371	58.0	32	74.4	1403	58.3	
1 child	657	27.8	7	16.3	664	27.6	
≥2 children	336	14.2	4	9.3	340	14.1	
**Smoking during pregnancy**	105	4.4	2	4.7	107	4.4	0.95
**Fertility preservation at diagnosis**^a^	94	4.0	23	53.5	117	4.9	<0.001
**Infertility after cancer diagnosis**	497	21.0	20	46.5	517	21.5	<0.001
**Mode of conception**							<0.001
Natural conception	2159	91.3	31	72.1	2190	91.0	
ART treatment	205	8.7	12	27.9	217	9.0	
**Gestational hypertension**	133	5.6	0	0.0	133	5.5	0.11
**Pre-eclampsia**	92	3.9	0	0.0	92	3.8	0.19
**Gestational diabetes mellitus**	28	1.2	1	2.3	29	1.2	0.50
**Placental abruption**	12	0.5	0	0.0	12	0.5	0.64
**Premature rupture of membranes**	72	3.0	2	4.7	74	3.1	0.55
**Induced delivery**	402	17.0	8	18.6	410	17.0	0.78
**Mode of delivery**							0.59
Unassisted vaginal	1607	68.0	29	67.4	1636	68.0	
Assisted vaginal	178	7.5	3	7.0	181	7.5	
Planned cesarean	276	11.7	3	7.0	279	11.6	
Emergency cesarean	303	12.8	8	18.6	311	12.9	
**Preterm birth <37 weeks**	207	8.8	5	11.6	212	8.8	0.51
**Multiple birth**	46	1.9	1	2.3	47	2.0	0.86
**Small for gestational age**^b^	58	2.5	0	0.0	58	2.4	0.58
**Large for gestational age**^c^	73	3.1	3	7.0	76	3.2	0.35
**Congenital anomaly**^d^	66	2.8	1	2.3	67	2.8	0.86
**Apgar <7 at 5 min**	55	2.3	2	4.7	57	2.4	0.32

Abbreviations: GnRHa = Gonadotropin-releasing hormone agonists, ART = assisted reproductive technology.

a Fertility preservation by using gonadotropins and GnRH antagonists at time of diagnosis.

b Birth weight below the 2.5th percentile for gestational age.

c Birth weight above the 97.5th percentile for gestational age.

d Congenital anomaly, ICD-10 codes Q00-Q99, registered in the Medical Birth Register.

## Discussion

At the time of performing this study, the proportion of patients prescribed with GnRHa in connection with cancer treatment in Sweden was unknown. Our study found a low prescription rate of GnRHa in Sweden, possibly explained by the established tax-financed regional programs for fertility preservation covering the whole country.^16^^,^^17^ Additionally, the Swedish guidelines do not include the use of GnRHa “off-label” for fertility preservation of women or teenage girls diagnosed with cancer,^18^ which has been in line with international guidelines.^10^^,^^11^ However, GnRHa co-treatment during chemotherapy for cancer with the aim of reducing chemotherapy-induced gonadotoxicity is empirically practiced worldwide and also in Sweden, and the medication can be prescribed by the treating oncologist or by a gynecologist.

Our study found no evidence of a higher childbirth rate among women who received GnRHa during cancer treatment, compared to women without GnRHa, after a mean follow-up of 4.5 years since diagnosis. We were able to consider several confounding factors including age, calendar period, previous parity, education level, country of birth and stage for breast cancer, relevant for the investigation of reproductive and oncologic outcomes. In particular, age and previous parity are known confounders for reproductive outcomes and most post-cancer births were achieved by women that were nulliparous at time of cancer diagnosis in this cohort. Women who received GnRHa co-treatment were more often nulliparous at time of cancer treatment, underwent more often procedures for fertility preservation aimed at oocyte or embryo cryopreservation and had more frequently post-cancer infertility diagnoses. These findings may suggest that they were more active in attempting pregnancy and in seeking reproductive healthcare than women who did not receive GnRHa. Although most childbirths in the cohort were naturally conceived, the use of medical treatments using ART to conceive was also more common in women who received GnRHa. These findings are in line with the report of higher frequency of pregnancy attempts in women who received GnRHa vs women who did not, which has been observed also in randomized unblinded studies of GnRHa in women with breast cancer.^7^^,^^19^ At present, none of the GnRHa trials has been double blinded or placebo-controlled, and all women who participated in these trials knew that they were receiving GnRHa medication that could potentially preserve their fertility, thus treatment assignment may have influenced active pregnancy attempts and the outcome of post-cancer fertility.^4^^,^^19^, ^20^, ^21^

There is currently a lack of large cohort studies with reliable follow-up of the women who have received GnRHa during cancer treatment. Our study contributes with the largest cohort to date and provides high quality outcome data with nationwide coverage.^22^ The Prescribed Drug Register is automatically updated every month with full coverage for all dispensations of prescription drugs at Swedish pharmacies since 2005 and is essentially complete for the prescription of GnRHa. The Medical Birth Register has a high coverage on 98.6% of all births occurring in Sweden,^14^ and linkages to the Cause of Death Register and the Migration Register enabled accurate and complete follow-up of all women in the cohort. The use of ART could also be identified with high reliability using the National Quality Registry for Assisted Reproduction.

Possible reasons for the discrepancy between our findings and those of previous clinical studies and meta-analyses include the heterogeneous surrogates of fertility used in previous studies and also their small sample size.^23^ The estimation of relevant live birth's outcome differences in interventional RCTs requires a sample size of about 1000–1300 women, all attempting pregnancy, to achieve the power to find differences between two treatment arms with respect to having a childbirth.^24^^,^^25^ The investigation of post-cancer fertility is complex, as women being treated for cancer are likely recommended to complete standard follow-up and delay family building, thus the *a priori* very large population needed for a clinical investigation of childbirth post-cancer is probably hard to achieve. However, we would expect to find statistically significant estimates in a large study, such as ours, if such fertility protective effects would exist. On the other hand, small scale RCTs would have the power to detect potential improvements in the ovarian reserve, longitudinally over time, but so far, no beneficial effect in women receiving GnRHa co-treatment during chemotherapy has been found.^8^^,^^9^^,^^26^^,^^27^

Our study is limited by the lack of information on pregnancy attempts or childbearing wish, however it provides information on how the pregnancies were achieved and if the women required fertility treatment using ART. Since GnRHa co-treatment might have been offered to women who desired future children, this may lead to estimates larger than the true association. In women with breast cancer who received GnRHa co-treatment, the mean follow-up time was 4.6 years from diagnosis and the mean age at end of follow-up was 38.5 years in our study. An additional shortcoming is the lack of information on subtypes of cancer and the type and dosage of chemotherapy. It is possible that GnRHa co-treatment was given to women at higher risk of infertility, which may bias results to the null. Women with estrogen positive breast cancers are often recommended adjuvant endocrine therapy for a minimum of five years, and during that time they should avoid conceiving. Among breast cancer patients who delivered a child, the mean time from diagnosis to delivery was 4.3 years in women who received adjuvant endocrine therapy and 2.7 years in women who did not. It is possible that a longer follow-up might be required to detect an effect on childbearing in that specific patient subgroup. At present, the long-term follow-up results of the POSITIVE study are highly awaited, to identify the women that could reduce the time on adjuvant endocrine treatment to achieve pregnancy on the short term while keeping relapse rates unaffected.^28^

To date, studies indicate that only a minority of women, between 10 and 20%, seek help to achieve pregnancy after cancer treatment.^17^^,^^29^, ^30^, ^31^ Hence, most women might not attempt pregnancy in the future. For this reason, there is a need to find further research methods to investigate appropriate outcomes for female fertility after cancer.

In this population-based cohort study on post-cancer childbirths, we found similar childbirth rates in women with and without GnRHa co-treatment, and thus we did not find evidence that use of GnRHa during cancer treatment is associated with improved or maintained fertility. A longer follow-up may be needed to capture post-cancer births, particularly in women that are recommended to complete long-term adjuvant endocrine therapy.

## Contributors

KARW, HK and FEL conceived and designed the study. FEL analyzed the data, KARW and FEL drafted the manuscript. All authors critically discussed the findings, revised the manuscript and finally approved it for submission.

## Data sharing statement

The data that support the findings of this study are available from register holders (Statistics Sweden, Swedish National Board of Health and Welfare and the National Quality Registry of Assisted Reproduction) for researchers with relevant ethical approvals and who meet the criteria for access to confidential data. The data are not publicly available due to restrictions by Swedish law, in order to protect patient privacy.

## Declaration of interests

KARW reports research funding provision by: The Swedish Cancer Society, The Swedish Research Council, the Nordic Cancer Union NCU; The Swedish Childhood Cancer Fund; The Radiumhemmets Forskningsfonder; Stockholm County Council and Karolinska Institutet supporting the research presented in this manuscript. She also reports grants from NovoNordisk Ferring and Merck pharmaceuticals that are not associated with this research. All grants have been given to the Karolinska Institutet and/or University Hospital. No personal payments. She has received consulting fees from the Swedish Ministry of Health and Welfare as expert in assisted reproduction and fertility preservation. She has received Honoraria from Roche, Pfizer, Merck.

ASO reports research funding by Karolinska Institutet KID and Forte and honoraria from Ferring pharmaceuticals and Abbott.

JB reports research grants from Amgen, AstraZeneca, Bayer, Merck, Pfizer, Roche and Sanofi-Aventis to Karolinska Institutet and/or University Hospital. No personal payments. He has received honoraria from Roche, AstraZeneca, Coronis, Asklepios Cancer Research AB and Stratipath AB.

The remaining authors do not report any conflicts of interest.
