# Supplementary material for: Gonadotropin Releasing Hormone agonist (GnRHa) during chemotherapy and post-cancer childbirths – a Nationwide population-based cohort study of 24,922 women diagnosed with cancer in Sweden
Source: eClinicalMedicine. 2023 Dec 7;67:102335. doi: 10.1016/j.eclinm.2023.102335 (PMC10837528; doi:10.1016/j.eclinm.2023.102335)
Supplement: Appendix [file mmc1.docx]

**WEB MATERIALS**

Gonadotropin Releasing Hormone agonist (GnRHa) during chemotherapy in women with cancer and post-cancer childbirths–

A Nationwide population-based cohort study

This supplemental material has been provided by the authors
to give readers additional information about their work.

**Webtable 1. ICD-10 codes used to categorize cancer types**

| Cancer site | ICD-10 codes |
| --- | --- |
| Digestive organs | C15-C26 |
| Respiratory and intrathoracic | C30-C39 |
| Bone and cartilage | C40-C41 |
| Skin | C43-C44 |
| Mesothelial and soft tissue | C45-C49 |
| Breast | C50 |
| Cervix | C53 |
| Other gynecological | C51-C52, C54-C58 |
| Urinary tract | C64-C68 |
| Central nervous system | C70-C72 |
| Endocrine glands | C73-C75 |
| Lymphoma | C81-C86 |
| *Hodgkin lymphoma* | *C81* |
| *Other lymphoma* | *C82-C86* |
| Leukemia | C90-C95 |
| Other cancers* | C00-C14, C69, C76-C80, C88, C96-C97 |

*No women diagnosed with cancer at these sites were prescribed GnRHa.

**Webtable 2. Characteristics of women diagnosed with lymphoma at ages 15-45 years in Sweden 2005-2017**

|  | Women without GnRHa (unexposed) | | Women with GnRHa (exposed) | | | Total | | | p-value | |
| --- | --- | --- | --- | --- | --- | --- | --- | --- | --- | --- |
|  | No. | % | No. | % | No. | | % |  | |  |
| Total number of patients | 1112 | 100·0 | 32 | 100·0 | 1144 | | 100·0 |  | |  |
| Type of lymphoma |  |  |  |  |  | |  | 0·001 | |  |
| Hodgkin lymphoma | 537 | 48·3 | 25 | 78·1 | 562 | | 49·1 |  | |  |
| Other lymphoma | 575 | 51·7 | 7 | 21·9 | 582 | | 50·9 |  | |  |
| Age at diagnosis |  |  |  |  |  | |  | 0·007 | |  |
| 15-19 years | 126 | 11·3 | 6 | 18·8 | 132 | | 11·5 |  | |  |
| 20-24 years | 147 | 13·2 | 7 | 21·9 | 154 | | 13·5 |  | |  |
| 25-29 years | 192 | 17·3 | 8 | 25·0 | 200 | | 17·5 |  | |  |
| 30-34 years | 196 | 17·6 | 9 | 28·1 | 205 | | 17·9 |  | |  |
| 35-39 years | 167 | 15·0 | 2 | 6·3 | 169 | | 14·8 |  | |  |
| 40-45 years | 284 | 25·5 | 0 | 0·0 | 284 | | 24·8 |  | |  |
| Country of birth |  |  |  |  |  | |  | 0·64 | |  |
| Nordic | 905 | 81·4 | 25 | 78·1 | 930 | | 81·3 |  | |  |
| Non-Nordic | 207 | 18·6 | 7 | 21·9 | 214 | | 18·7 |  | |  |
| Parity at diagnosis |  |  |  |  |  | |  | 0·013 | |  |
| 0 (nulliparous) | 550 | 49·5 | 23 | 71·9 | 573 | | 50·1 |  | |  |
| 1 child | 190 | 17·1 | 6 | 18·8 | 196 | | 17·1 |  | |  |
| ≥2 children | 372 | 33·5 | 3 | 9·4 | 375 | | 32·8 |  | |  |
| Year of diagnosis |  |  |  |  |  | |  | 0·10 | |  |
| 2005-2008 | 320 | 28·8 | 7 | 21·9 | 327 | | 28·6 |  | |  |
| 2009-2011 | 269 | 24·2 | 6 | 18·8 | 275 | | 24 |  | |  |
| 2012-2014 | 330 | 29·7 | 8 | 25·0 | 338 | | 29·5 |  | |  |
| 2015-2017 | 193 | 17·4 | 11 | 34·4 | 204 | | 17·8 |  | |  |
| Education before diagnosis |  |  |  |  |  | |  | 0·53 | |  |
| Compulsory school | 182 | 16·4 | 3 | 9·4 | 185 | | 16·2 |  | |  |
| Secondary school | 415 | 37·3 | 13 | 40·6 | 428 | | 37·4 |  | |  |
| Higher education <3y | 141 | 12·7 | 2 | 6·3 | 143 | | 12·5 |  | |  |
| Higher education ≥3y | 283 | 25·4 | 10 | 31·3 | 293 | | 25·6 |  | |  |
| Missing | 91 | 8·2 | 4 | 12·5 | 95 | | 8·3 |  | |  |
| Fertility preservation* | 106 | 9·5 | 17 | 53·1 | 123 | | 10·8 | <0·001 | |  |

*Using gonadotropins and GnRH antagonist.

Abbreviations: GnRHa = Gonadotropin-releasing hormone agonist.

**Webtable 3. Characteristics of women diagnosed with other cancer than breast or lymphoma at ages 15-45 years in Sweden 2005-2017**

|  | Women without GnRHa (unexposed) | | Women with GnRHa (exposed) | | Total | | p-value |
| --- | --- | --- | --- | --- | --- | --- | --- |
|  | No. | % | No. | % | No. | % |  |
| Total number of patients | 16 788 | 100·0 | 44 | 100·0 | 16 832 | 100·0 |  |
| Cancer site |  |  |  |  |  |  | <0·001 |
| Skin | 3969 | 23·6 | 1 | 2·3 | 3970 | 23·6 |  |
| Endocrine glands | 3352 | 20·0 | 8 | 18·2 | 3360 | 20·0 |  |
| Cervix | 2562 | 15·3 | 7 | 15·9 | 2569 | 15·3 |  |
| Other gynecological | 1254 | 7·5 | 2 | 4·5 | 1256 | 7·5 |  |
| Digestive organs | 1724 | 10·3 | 6 | 13·6 | 1730 | 10·3 |  |
| Central nervous system | 1609 | 9·6 | 2 | 4·5 | 1611 | 9·6 |  |
| Leukemia | 582 | 3·5 | 8 | 18·2 | 590 | 3·5 |  |
| Respiratory and intrathoracic | 406 | 2·4 | 4 | 9·1 | 410 | 2·4 |  |
| Urinary tract | 335 | 2·0 | 2 | 4·5 | 337 | 2·0 |  |
| Mesothelial and soft tissue | 274 | 1·6 | 1 | 2·3 | 275 | 1·6 |  |
| Bone and cartilage | 109 | 0·6 | 3 | 6·8 | 112 | 0·7 |  |
| Other cancers | 612 | 3·6 | 0 | 0·0 | 612 | 3·6 |  |
| Age at diagnosis |  |  |  |  |  |  | <0·001 |
| 15-19 years | 604 | 3·6 | 3 | 6·8 | 607 | 3·6 |  |
| 20-24 years | 1108 | 6·6 | 6 | 13·6 | 1114 | 6·6 |  |
| 25-29 years | 1896 | 11·3 | 13 | 29·5 | 1909 | 11·3 |  |
| 30-34 years | 2744 | 16·3 | 14 | 31·8 | 2758 | 16·4 |  |
| 35-39 years | 3751 | 22·3 | 5 | 11·4 | 3756 | 22·3 |  |
| 40-45 years | 6685 | 39·8 | 3 | 6·8 | 6688 | 39·7 |  |
| Country of birth |  |  |  |  |  |  | 0·69 |
| Nordic | 14 125 | 84·1 | 38 | 86·4 | 14 163 | 84·1 |  |
| Non-Nordic | 2663 | 15·9 | 6 | 13·6 | 2669 | 15·9 |  |
| Parity at diagnosis |  |  |  |  |  |  | <0·001 |
| 0 (nulliparous) | 6161 | 36·7 | 35 | 79·5 | 6196 | 36·8 |  |
| 1 child | 3016 | 18·0 | 5 | 11·4 | 3021 | 17·9 |  |
| ≥2 children | 7611 | 45·3 | 4 | 9·1 | 7615 | 45·2 |  |
| Year of diagnosis |  |  |  |  |  |  | 0·31 |
| 2005-2008 | 4348 | 25·9 | 8 | 18·2 | 4356 | 25·9 |  |
| 2009-2011 | 4196 | 25·0 | 9 | 20·5 | 4205 | 25·0 |  |
| 2012-2014 | 4619 | 27·5 | 13 | 29·5 | 4632 | 27·5 |  |
| 2015-2017 | 3625 | 21·6 | 14 | 31·8 | 3639 | 21·6 |  |
| Education before diagnosis |  |  |  |  |  |  | 0·98 |
| Compulsory school | 1898 | 11·3 | 6 | 13·6 | 1904 | 11·3 |  |
| Secondary school | 7129 | 42·5 | 17 | 38·6 | 7146 | 42·5 |  |
| Higher education <3y | 2390 | 14·2 | 7 | 15·9 | 2397 | 14·2 |  |
| Higher education ≥3y | 4622 | 27·5 | 12 | 27·3 | 4634 | 27·5 |  |
| Missing | 749 | 4·5 | 2 | 4·5 | 751 | 4·5 |  |
| Fertility preservation* | 107 | 0·6 | 16 | 36·4 | 123 | 0·7 | <0·001 |

*Using gonadotropins and GnRH antagonist.

Abbreviations: GnRHa = Gonadotropin-releasing hormone agonist.

**Webtable 4. Rates of childbirth, infertility and mortality in women diagnosed with cancer at ages 15-45 and who were nulliparous at diagnosis**

|  | With GnRHa | | No GnRHa (ref) | | Model 1 | Model 2 | Model 3 |
| --- | --- | --- | --- | --- | --- | --- | --- |
|  | **Events** | **PY** | **Events** | **PY** | **HR (95% CI)** | **HR (95% CI)** | **HR (95% CI)** |
| Breast cancer |  |  |  |  |  |  |  |
| Any childbirth | 22 | 530 | 76 | 6518 | 1·68 (1·01-2·78) | 1·54 (0·92-2·58) | 1·46 (0·86-2·47) |
| NC childbirth | 16 | 530 | 62 | 6518 | 1·46 (0·82-2·61) | 1·35 (0·75-2·45) | 1·31 (0·72-2·38) |
| Infertility | 19 | 489 | 49 | 6378 | 2·18 (1·24-3·83) | 2·25 (1·26-4·00) | 2·24 (1·25-4·04) |
| Mortality | 10 | 591 | 156 | 6804 | 0·75 (0·38-1·47) | 0·81 (0·41-1·58) | 0·76 (0·39-1·49) |
| Lymphoma |  |  |  |  |  |  |  |
| Any childbirth | 6 | 76 | 126 | 2497 | 1·87 (0·72-4·88) | 1·80 (0·57-4·84) | ·· |
| NC childbirth | 6 | 76 | 115 | 2497 | 2·00 (0·76-5·27) | 1·92 (0·71-5·17) | ·· |
| Infertility | 2 | 71 | 47 | 2365 | 1·69 (0·33-8·56) | 1·61 (0·30-8·81) | ·· |
| Mortality | 0 | 112 | 18 | 2984 | NA | NA | ·· |
| Other cancer |  |  |  |  |  |  |  |
| Any childbirth | 4 | 101 | 1169 | 23 754 | 0·80 (0·29-2·23) | 0·97 (0·35-2·73) | ·· |
| NC childbirth | 2 | 101 | 1021 | 23 754 | 0·44 (0·11-1·83) | 0·53 (0·13-2·20) | ·· |
| Infertility | 4 | 86 | 410 | 22 713 | 1·91 (0·65-5·66) | 1·96 (0·64-5·99) | ·· |
| Mortality | 3 | 125 | 475 | 28 356 | 0·85 (0·24-2·97) | 0·58 (0·15-2·18) | ·· |

Model 1 stratified by age at diagnosis and cancer type, adjusted for time since diagnosis.

Model 2 further adjusted for country of birth, education level, and calendar period.

Model 3 further adjusted for cancer stage at diagnosis.

Abbreviations: GnRHa = Gonadotropin-releasing hormone agonist, NC = natural conception.

**Webtable 5. Rates of childbirth, infertility and mortality in women diagnosed with cancer at ages 15-39 years in Sweden 2005-2017**

|  | With GnRHa | | No GnRHa (ref) | | Model 1 | Model 2 | Model 3 |
| --- | --- | --- | --- | --- | --- | --- | --- |
|  | **Events** | **PY** | **Events** | **PY** | **HR (95% CI)** | **HR (95% CI)** | **HR (95% CI)** |
| Breast cancer |  |  |  |  |  |  |  |
| Any childbirth | 26 | 770 | 161 | 12 091 | 1·54 (1·00-2·37) | 1·16 (0·75-1·81) | 1·14 (0·73-1·77) |
| NC childbirth | 19 | 770 | 143 | 12 091 | 1·24 (0·76-2·03) | 0·99 (0·60-1·64) | 0·97 (0·59-1·61) |
| Infertility | 23 | 727 | 62 | 11 921 | 3·92 (2·35-6·54) | 2·21 (1·31-3·71) | 2·24 (1·32-3·79) |
| Mortality | 19 | 847 | 307 | 12 726 | 0·88 (0·55-1·42) | 0·95 (0·59-1·55) | 0·97 (0·60-1·59) |
| Lymphoma |  |  |  |  |  |  |  |
| Any childbirth | 8 | 99 | 207 | 3693 | 1·35 (0·64-2·86) | 1·24 (0·58-2·65) | ·· |
| NC childbirth | 8 | 99 | 193 | 3693 | 1·49 (0·70-3·18) | 1·39 (0·65-2·99) | ·· |
| Infertility | 2 | 94 | 59 | 3524 | 1·41 (0·31-6·35) | 1·28 (0·26-6·29) | ·· |
| Mortality | 0 | 142 | 23 | 4522 | NA | NA | ·· |
| Other cancer |  |  |  |  |  |  |  |
| Any childbirth | 7 | 126 | 1955 | 40 095 | 1·29 (0·59-2·82) | 1·31 (0·60-2·86) | ·· |
| NC childbirth | 3 | 126 | 1791 | 40 095 | 0·56 (0·17-1·78) | 0·59 (0·18-1·89) | ·· |
| Infertility | 5 | 110 | 481 | 38 889 | 2·56 (0·94-6·96) | 2·46 (0·90-6·68) | ·· |
| Mortality | 5 | 168 | 691 | 48 071 | 0·75 (0·29-1·96) | 0·76 (0·29-1·99) | ·· |

Model 1 stratified by age at diagnosis and cancer type, adjusted for time since diagnosis.

Model 2 further adjusted for country of birth, education level, calendar period, and parity before diagnosis.

Model 3 further adjusted for cancer stage at diagnosis.

Abbreviations: GnRHa = Gonadotropin-releasing hormone agonist, NC = natural conception.

**Webtable 6. Rates of childbirth, infertility and mortality in women diagnosed with breast cancer at ages 15-45 years in Sweden 2005-2017, stratified by adjuvant endocrine therapy**

|  | **With GnRHa** | | **No GnRHa (ref)** | | **Model 1** | **Model 2** | **Model 3** |
| --- | --- | --- | --- | --- | --- | --- | --- |
|  | **Events** | **PY** | **Events** | **PY** | **HR (95% CI)** | **HR (95% CI)** | **HR (95% CI)** |
| **Breast cancer with adjuvant endocrine therapy** |  |  |  |  |  |  |  |
| Childbirth | 14 | 637 | 49 | 21 169 | 2·66 (1·40-5·05) | 1·78 (0·90-3·54) | 1·56 (0·77-3·16) |
| NC childbirth | 11 | 637 | 44 | 21 169 | 2·41 (1·19-4·90) | 1·69 (0·79-3·59) | 1·49 (0·69-3·23) |
| Infertility | 13 | 613 | 39 | 21 064 | 3·37 (1·71-6·65) | 2·06 (1·02-4·15) | 2·12 (1·00-4·50) |
| Cancer mortality | 9 | 679 | 307 | 21 326 | 0·76 (0·38-1·53) | 0·71 (0·34-1·48) | 0·93 (0·44-1·97) |
| **Breast cancer without adjuvant endocrine therapy** |  |  |  |  |  |  |  |
| Childbirth | 14 | 438 | 119 | 11 953 | 1·53 (0·85-2·75) | 1·18 (0·63-2·21) | 1·12 (0·55-2·26) |
| NC childbirth | 9 | 438 | 104 | 11 953 | 1·02 (0·50-2·08) | 0·82 (0·38-1·78) | 0·76 (0·32-1·78) |
| Infertility | 11 | 416 | 25 | 11 877 | 6·76 (3·09-14·82) | 4·44 (1·74-11·32) | 5·78 (1·98-16·88) |
| Cancer mortality | 19 | 478 | 327 | 12 464 | 1·36 (0·84-2·19) | 1·35 (0·83-2·20) | 1·02 (0·58-1·78) |

Model 1 stratified by age at diagnosis and adjusted for time since diagnosis.

Model 2 further adjusted for country of birth, education level, calendar period, and parity before diagnosis.

Model 3 further adjusted for cancer stage at diagnosis.

Abbreviations: GnRHa = Gonadotropin-releasing hormone agonist, NC = natural conception.

**Webtable 7. Characteristics at first post-diagnosis delivery in women diagnosed with breast cancer at ages 15-45 years**

|  | Women without GnRHa (unexposed) | | Women with GnRHa (exposed) | | Total | | | p-value | |
| --- | --- | --- | --- | --- | --- | --- | --- | --- | --- |
|  | No. | % | No. | % | No. | % |  | |  |
| Women with live birth | 168 | 100·0 | 28 | 100·0 | 196 | 100·0 |  | |  |
| Age at time of delivery |  |  |  |  |  |  | 0·69 | |  |
| 20-29 years | 9 | 5·4 | 2 | 7·1 | 11 | 5·6 |  | |  |
| 30-39 years | 117 | 69·6 | 21 | 75·0 | 138 | 70·4 |  | |  |
| ≥40 years | 42 | 25·0 | 5 | 17·9 | 47 | 24·0 |  | |  |
| Time between cancer diagnosis and birth |  |  |  |  |  |  | 0·95 | |  |
| <2 years | 28 | 16·7 | 4 | 14·3 | 32 | 16·3 |  | |  |
| 2-4 years | 93 | 55·4 | 16 | 57·1 | 109 | 55·6 |  | |  |
| ≥5 years | 47 | 28·0 | 8 | 28·6 | 55 | 28·1 |  | |  |
| Previous parity |  |  |  |  |  |  | 0·005 | |  |
| 0 (nulliparous) | 76 | 45·2 | 22 | 78·6 | 98 | 50·0 |  | |  |
| 1 child | 57 | 33·9 | 4 | 14·3 | 61 | 31·1 |  | |  |
| ≥2 children | 35 | 20·8 | 2 | 7·1 | 37 | 18·9 |  | |  |
| Smoking during pregnancy | 4 | 2·4 | 1 | 3·6 | 5 | 2·6 | 0·71 | |  |
| Fertility preservation at diagnosis* | 47 | 28·0 | 17 | 60·7 | 64 | 32·7 | <0·001 | |  |
| Infertility after diagnosis | 35 | 20·8 | 16 | 57·1 | 51 | 26·0 | <0·001 | |  |
| Mode of conception |  |  |  |  |  |  | 0·020 | |  |
| Natural conception | 148 | 88·1 | 20 | 71·4 | 168 | 85·7 |  | |  |
| ART treatment | 20 | 11·9 | 8 | 28·6 | 28 | 14·3 |  | |  |
| Gestational hypertension | 5 | 3·0 | 0 | 0·0 | 5 | 2·6 | 0·36 | |  |
| Pre-eclampsia | 3 | 1·8 | 0 | 0·0 | 3 | 1·5 | 0·48 | |  |
| Gestational diabetes mellitus | 1 | 0·6 | 0 | 0·0 | 1 | 0·5 | 0·68 | |  |
| Placental abruption | 2 | 1·2 | 0 | 0·0 | 2 | 1·0 | 0·56 | |  |
| Premature rupture of membranes | 4 | 2·4 | 2 | 7·1 | 6 | 3·1 | 0·18 | |  |
| Induced delivery | 32 | 19·0 | 6 | 21·4 | 38 | 19·4 | 0·77 | |  |
| Mode of delivery |  |  |  |  |  |  | 0·13 | |  |
| Unassisted vaginal | 105 | 62·5 | 16 | 57·1 | 121 | 61·7 |  | |  |
| Assisted vaginal | 14 | 8·3 | 3 | 10·7 | 17 | 8·7 |  | |  |
| Planned cesarean | 25 | 14·9 | 1 | 3·6 | 26 | 13·3 |  | |  |
| Emergency cesarean | 24 | 14·3 | 8 | 28·6 | 32 | 16·3 |  | |  |
| Preterm birth (<37 gestational weeks) | 10 | 6·0 | 5 | 17·9 | 15 | 7·7 | 0·028 | |  |
| Multiple birth | 3 | 1·8 | 1 | 3·6 | 4 | 2·0 | 0·54 | |  |
| Small for gestational age† | 3 | 1·8 | 0 | 0·0 | 3 | 1·5 | 0·65 | |  |
| Large for gestational age‡ | 6 | 3·6 | 1 | 3·6 | 7 | 3·6 | 0·83 | |  |
| Congenital anomaly§ | 2 | 1·2 | 1 | 3·6 | 3 | 1·5 | 0·34 | |  |
| Apgar <7 at 5 minutes | 3 | 1·8 | 2 | 7·1 | 5 | 2·6 | 0·10 | |  |

* Fertility preservation using gonadotropins and GnRH antagonists a time of diagnosis.

† Birth weight below the 2·5^th^ percentile for gestational age.

‡ Birth weight above the 97·5^th^ percentile for gestational age.

§ Congenital anomaly, ICD-10 codes Q00-Q99, registered in the Medical Birth Register.

Abbreviations: GnRHa = Gonadotropin-releasing hormone agonist, ART = assisted reproductive technology.
